# Prognostic Impact of H19/Cell Adhesion Molecules Circuitry on Prostate Cancer Biopsy

**DOI:** 10.3390/biomedicines12102322

**Published:** 2024-10-12

**Authors:** Valeria Pecci, Francesco Pierconti, Angela Carlino, Francesco Pinto, Ugo Gradilone, Sara De Martino, Dante Rotili, Claudio Grassi, Alfredo Pontecorvi, Carlo Gaetano, Lidia Strigari, Antonella Farsetti, Simona Nanni

**Affiliations:** 1 Department of Translational Medicine and Surgery, Università Cattolica del Sacro Cuore, 00168 Rome, Italy; valeria.pecci@unicatt.it (V.P.); francesco.pinto@unicatt.it (F.P.); ugo.gradilone@gmail.com (U.G.); alfredo.pontecorvi@unicatt.it (A.P.); 2 Fondazione “Policlinico Universitario A. Gemelli IRCCS”, 00168 Rome, Italy; francesco.pierconti@unicatt.it (F.P.); ange.carlino@gmail.com (A.C.); claudio.grassi@unicatt.it (C.G.); 3 Department of Woman, Child and Public Health, Università Cattolica del Sacro Cuore, 00168 Rome, Italy; 4 National Research Council (CNR)–Istituto di Analisi dei Sistemi ed Informatica “Antonio Ruberti” (IASI), 00185 Rome, Italy; sara.demartino@iasi.cnr.it; 5 Dipartimento di Chimica e Tecnologie del Farmaco, Sapienza Università di Roma, 00185 Rome, Italy; dante.rotili@uniroma1.it; 6 Department of Neuroscience, Università Cattolica del Sacro Cuore, 00168 Rome, Italy; 7 Laboratory of Epigenetics, Istituti Clinici Scientifici Maugeri IRCCS, 27100 Pavia, Italy; carlo.gaetano@icsmaugeri.it; 8 Department of Medical Physics, S. Orsola Malpighi University Hospital, 40138 Bologna, Italy; lidia.strigari@aosp.bo.it

**Keywords:** prostate cancer, molecular biomarkers, prognosis, patient’s stratification

## Abstract

Introduction: Metastatic prostate cancer (PCa) presents a significant challenge in oncology due to its high mortality rate and the absence of effective biomarkers for predicting patient outcomes. Building on previous research that highlighted the critical role of the long noncoding RNA (lncRNA) H19 and cell adhesion molecules in promoting tumor progression under hypoxia and estrogen stimulation, this study aimed to assess the potential of these components as prognostic biomarkers for PCa at the biopsy stage. Methods: This research utilized immunohistochemistry and droplet digital PCR to analyze formalin-fixed paraffin-embedded (FFPE) biopsies, focusing on specific markers within the H19/cell adhesion molecules pathway. Results: A novel multivariate analysis led to a “BioScore”, a composite biomarker score to predict disease progression. This score is based on evaluating five key markers: the expression levels of Hypoxia-Inducible Factor 2 Alpha (HIF-2α), endothelial Nitric Oxide Synthase (eNOS), β4 integrin, E-cadherin transcript (CDH1), and lncRNA H19. The criteria for the “BioScore” involve identifying three out of these five markers, combining elevated levels of HIF-2α, eNOS, β4 integrin, and CDH1 with reduced H19 expression. Conclusions: This finding suggests the possibility of identifying, at the time of biopsy, PCa patients at higher risk of metastasis based on dysregulation in the H19/cell adhesion molecules circuitry. This study provides a valuable opportunity for early intervention in managing PCa, potentially contributing to personalized treatment strategies.

## 1. Introduction

Prostate cancer is the second most prevalent male cancer in the world, just behind lung cancer, with a 4% to 6% annual increase in advanced disease over the past decade [1,2]. Radical prostatectomy and radiotherapy represent standard therapies with good results in terms of 5-year survival rate for localized prostate cancer [3]. However, about 20–40% of clinically localized PCa still develops metastatic disease despite curative treatment attempts, with recent trends showing an increasing incidence [1,4,5].

Notably, no effective biomarkers exist to identify patients who will develop metastatic disease at the time of biopsy or during follow-up. Recent research suggests a multimodal approach to the diagnosis and management of prostatic carcinoma, involving multiparametric magnetic resonance imaging (mpMRI), prostate-specific membrane antigen (PSMA) positron emission tomography (PET/CT), and the fusion-guided prostatic biopsy technique [6]. In this regard, PCa is predominantly detected by a widely used prostate-specific antigen (PSA) and its derivates blood test. Other blood-based biomarkers (PHI/4K score/IsoPSA) [7] and urine biomarkers (PCA3/SelectMDX/Mi Prostate score (MiPS)/ExoDX) [8,9] have been introduced in the last decade with not significant effect. Multiparametric magnetic resonance imaging (mpMRI) is now an important aspect of the diagnostic pathway in prostate cancer, improving the detection of clinically significant prostate cancer, enabling accurate localization of appropriate sites to biopsy, and reducing unnecessary biopsies in most patients with normal magnetic resonance imaging scans. When MRI is positive (i.e., PI-RADS > 3), combined targeted and systematic biopsy is the standard for definitive diagnosis [10,11]. Moreover, in 2016, the International Society of Uro-Pathology (ISUP), starting from Gleason score, suggested a new stratification of the risk of the prostate cancer in five groups, in order to better identify tumors with high risk for local recurrence, involvement of lymph nodes, and metastasis [12].

Routine diagnosis is made in most cases on H&E-stained slides from biopsy, with particular attention to the evaluation of biopsy-proven Gleason score that in almost 17% of the cases was underestimated at radical prostatectomy [13]. Current recurrence risk prediction lies in clinicopathological characteristics, such as PSA, Gleason score, and pathological T stage, as well as in conventional imaging like abdominal/pelvic magnetic resonance imaging [14,15]. Although efforts have been made to obtain a more accurate prognostic stratification of PCa patients, we still have no resolution, mostly in patients of clinical intermediate risk (ISUP 2-3), who represent almost all patients analyzed in the present cohort. The need for immunohistochemical biomarkers to assess recurrence risk following radical prostatectomy remains unresolved [16]. It has become increasingly clear that a single biomarker is not sufficient to meet outcome prediction, and the current approaches rely on combining several markers with next-generation sequencing (NGS) or deep learning techniques ([17,18,19] and references therein). Therefore, identifying patients at high risk of recurrence represents a high priority in the field. Here, we focused on developing an individualized approach for early prediction of patient outcomes.

Specifically, we centered on a key metastatic process known as cohesive metastatic phenotype or collective cell migration, in which cohesive clusters of cells invade surrounding tissues [20]. Cohesive clusters of cells are characterized by cell–cell adhesions (e.g., the presence of E-cadherin and intermediate filament proteins) as well as by integrin-mediated adhesion to the extracellular matrix (ECM) [20]. Our previous studies in PCa revealed that estrogen signaling and hypoxia signaling act synergistically in regulating gene transcription towards an aggressive phenotype, from a mechanistic point of view, by forming a combinatorial complex with endothelial Nitric Oxide Synthase/Estrogen Receptor β/Hypoxia-Inducible Factor 2 Alpha (eNOS/ERβ/HIF-2α) on the promoter of target genes [21,22,23,24]. We previously showed (i) a transcriptional down-regulation of lncRNA H19 under combined pro-tumoral stimuli, i.e., estrogen and hypoxia, that, in turn, induced both E-cadherin and β4 integrin expression, thus eliciting a cohesive metastatic phenotype, and (ii) the in vivo role of this H19/cell adhesion molecules circuitry in promoting metastasis in bones, lungs, and the liver [24,25]. Notably, the role of cell adhesion molecules in the cohesive metastatic phenotype was recently discovered specifically for prostate cancer, in which a cluster of tumor cells move together across a two-dimensional layer of extracellular matrix while retaining their cell–cell junction [24,25,26,27]. Accordingly, cadherin and integrin are considered promising targets for cancer therapy with potential values in metastatic disease [27,28,29,30]. Of note, HIF-2α expression was associated with Gleason score and ISUP grade in PCa radical prostatectomy specimens [31]. HIF-2α was also reported as an independent prognostic factor for poor overall survival in clear-cell renal cell carcinoma [32]. Noteworthy, higher expression of eNOS, together with other oxidative stress markers, is associated with a lower survival rate in patients with pancreatic cancer [33]. Moreover, evidence recently emerged regarding the use of long noncoding RNA (lncRNA) as promising biomarkers for diagnosis and prognosis in PCa, also evaluated on exosomes or urine samples [34,35,36,37]. Intriguingly, the lncRNA H19 was found to be significantly elevated in serum extracellular vesicles of androgen-resistant PCa patients by droplet digital PCR (ddPCR) [38].

Our working hypothesis is that patients developing metastasis exhibit a tumor micro-environment characterized by altered estrogen and hypoxia signaling that activates the H19/cell adhesion molecules circuitry, governing the metastatic program toward a collective cancer cell migration. To validate the clinical relevance of the components of the H19/cell adhesion molecules circuitry in stratifying and classifying PCa patients, we analyzed an independent retrospective cohort of PCa biopsies. Our results underscored the potential of these components as novel prognostic indicators of PCa recurrence at the time of biopsy.

## 2. Materials Methods

### 2.1. Antibodies

β4 integrin (Abcam, Cambridge, UK, #ab133682, RRID: AB_2923284, and 450-11A, RRID: AB_396065, as in [24]), E-cadherin (GeneTex, Alton Pkwy Irvine, CA, USA, Cat# GTX100443, RRID: AB_10729586 and Abcam, #ab231303, RRID: AB_2923285), eNOS (BD Biosciences San Jose, CA, Cat# 610296, RRID: AB_397690), and HIF-2α (Abcam Cat# ab8365, RRID: AB_306519).

### 2.2. Ethics Approval and Consent to Participate

The ethical committee of IRCCS authorized patient enrollment and organotypic slice cultures (Fondazione Policlinico Gemelli-Università Cattolica of Rome, Italy (Protocol number: 23293/18; ID: 2133; date of approval: 21 June 2018)) and written informed consent was obtained from each patient in the presence of a physician providing explanation. All procedures were conducted according to the principles expressed in the Declaration of Helsinki, institutional regulation, and Italian laws and guidelines. 

### 2.3. Data Set Biopsy

A total of 50 prostatic biopsies with a diagnosis of acinar adenocarcinoma were performed at our institution and examined by two different pathologists (F.P. (Francesco Pierconti) and A.C.). All the analyzed data were collected as part of our institution’s routine diagnosis and treatment procedures, including minimum biopsy core length 12 mm [13,39]. The Gleason grade was assigned based on strictly classical (1977) and modified criteria (2005 and 2014 ISUP), and for cases in which consensus among the pathologists could not be agreed, a third pathologist was consulted to reach group consensus [12]. All the radical prostatectomies were examined and reviewed based on the protocol described by Montironi et al. in 2002, using whole-mount sections [40]. Pathological staging was performed according to the eighth edition of the TNM (Tumor, Node, Metastasis) Classification of Malignant Tumors [41].

### 2.4. Histological Analysis

**Figure 1:**
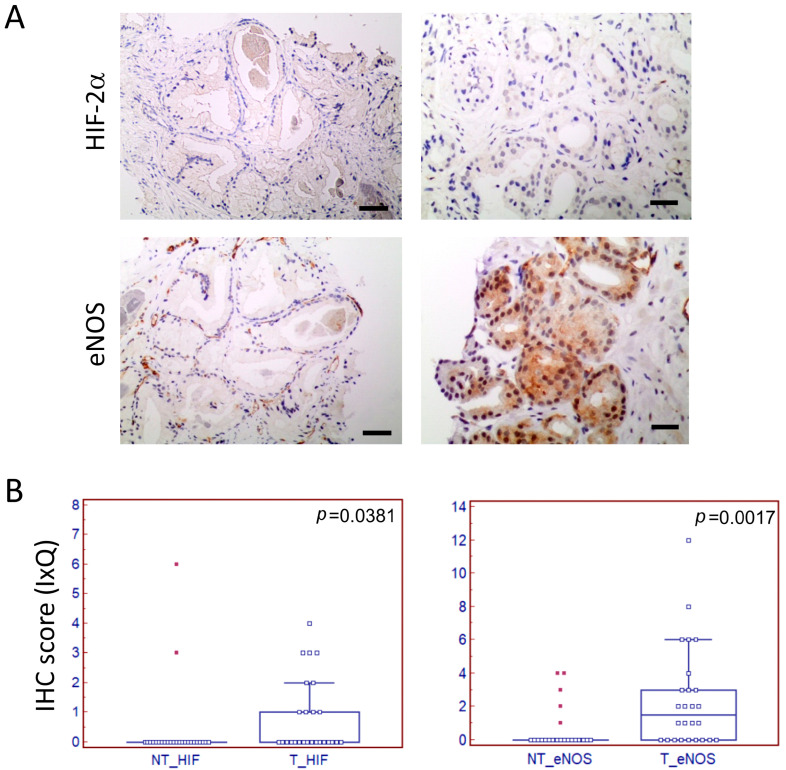
IHC on PCa biopsies and score in the normal and tumoral areas. (**A**) Representative IHC staining of PCa biopsies with a specific antibody to HIF2α and eNOS in normal (left, scale bar = 84 mm) and tumoral (right, scale bar = 210 mm) areas. The scale bar is shown as a black line. (**B**) The IHC score was evaluated as Intensity × Quantity (IxQ) in normal (NT) and tumoral (T) areas for eNOS and HIF-2α staining. *p* values are indicated.

**Figure 2:**
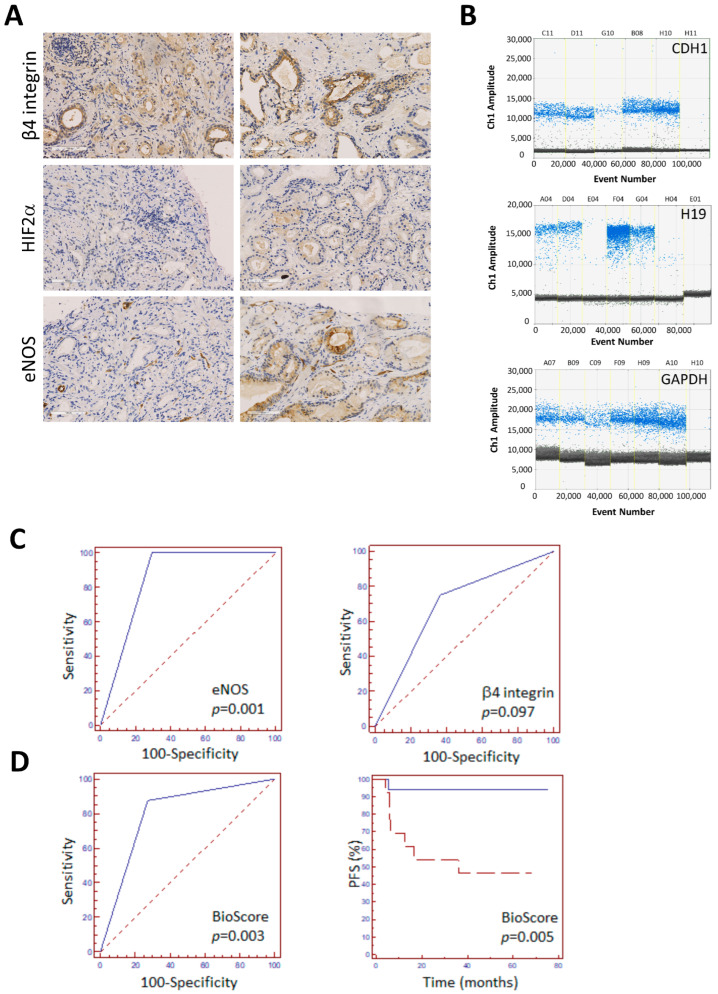
Analysis of PCa biopsies and identification of prognostic score based on combinatorial expression of biomarkers. (**A**) Representative IHC staining of PCa biopsies with antibodies to β4 integrin, HIF2α, and eNOS. Scale bar: 100 μm. (**B**) Representative gene expression analysis by ddPCR of CDH1, H19, and control gene GAPDH on PCa biopsies and negative controls. (**C**) ROC curve analysis of PFS using eNOS and β4 integrin expression on PCa biopsies. The dashed line represents the random assignment AUC and 95% confidence interval (CI) details in Table 2. (**D**) Left: ROC curve analysis of PFS using the novel “BioScore” determined on PCa biopsies (cut-off and AUC details in Table 2). Right: Kaplan–Meier curve of PFS for patients with low or high “BioScore” levels (dashed and solid line, respectively). The Hazard Ratio with 95% CI was 0.090 (0.031–0.532). *p* values are indicated.

For histological and immunohistochemical analyses, tissues were fixed in 10% formalin (Thermo Fisher Scientific, Waltham, MA, USA). Unstained tissue sections (4 μm thick) were cut from formalin-fixed, paraffin-embedded blocks and mounted on a positively charged glass slide. IHC was performed using the Leica Bond (LBO, Milan, Italy) immunostainer or a Ventana Benchmark XT automated immunostainer (Ventana Medical Systems, Tucson, AZ, USA). The following tests were performed: HIF2α (clone ep190b, Abcam; 1:100 dilution, epitope retrieval (ER) pH6 for 10 min), anti-β4-integrin (clone 450-11A, 1:100 dilution, ER pH6 for 20 min), anti-eNOS (clone 3/eNOS/NOS Type III Cat.#610296, BD Biosciences; 1:250 dilution, ER pH6 for 20 min), and cytokeratin AE1/AE3 (Agilent DAKO, Santa Clara, CA, USA), #GA053, ER pH6 for 20 min). Hematoxylin was used for nuclear counterstaining. Quantification of IHC staining was performed as in [23]. Briefly, the final score was assessed individually on normal and tumoral areas as Intensity × Quantity [staining Intensity = 0 (negative), 1 (low), 2 (medium), 3 (high); Quantity = 0 (negative), 1 (1–9% positive cells), 2 (10–39% positive cells), 3 (40–69% cells), 4 (70–100% positive cells)]. Tissue specimens were examined using the Olympus cellSens platform and a SC30 microscope with Olympus cellSens Entry software version 2.3 for digital image acquisition (images shown in Figure 1 and Figure S2). Whole-slide imaging (WSI) was performed using NanoZoomer 2.ORS (Hamamatsu Photonics, Hamamatsu, Japan) using 20× magnification (0.46 microns/pixel), with the images shown in Figure 2.

### 2.5. RNA Extraction, cDNA Preparation, Real-Time PCR, and Droplet Digital PCR

RNA from FFPE biopsies was extracted using RNeasy FFPE Kit (Qiagen, Hilden, Germany, #73504) on the QIACUBE HT system (Qiagen). cDNA preparation, preAmp PCR, and ddPCR were performed on the QX200 droplet digital PCR system (Biorad, Milan, Italy) as described in [42]. Briefly, cDNA preparation was performed using the high-capacity kit (Applied Biosystems, Foster City, CA, USA) according to the instructions. A preAmp step was performed using 2 μL of cDNA, EvaGreen mix reaction, and specific primers at 40 nM for 14 cycles at 95 °C for 15 s and at 58 °C for 4 min. One microliter of the preAmp mix (1:10 dilution) was used to perform ddPCR using EvaGreen (total droplet number > 12,000). A representative detection of lncRNA and mRNA in PCa biopsy is shown in Figure S1. Gene quantification was in copy numbers/microliter. The target RNA level was normalized to the housekeeping gene. ddPCR was performed using EvaGreen at 60 °C for an annealing/extension step according to the manufacturer’s instructions on a QX-200 instrument (Bio-Rad) with the following primers at the indicated final concentrations: sequence of primers to H19 (200 nM), CDH1 (200 nM), ITGB4 (100 nM), P0 (200 nM), and GAPDH (100 nM) were as in [24].

### 2.6. Statistical Analysis

Statistical differences between groups (e.g., tumors and normal areas) were analyzed by the paired Student *t*-test with a degree of significance (*p*) of less than 0.05. The impact of each biomarker was analyzed by both univariate and multivariate Cox analyses (UVA and MVA, respectively). Regarding UVA, the observed progression was used as “truth”, i.e., the gold standard for nonparametric clustered receiver operating characteristic (ROC) analysis, to evaluate the utility of each investigated biomarker. By comparing the observed progression and the value of each biomarker, the True Positive Ratio (sensitivity) and the False Positive Ratio (1-specificity) were plotted in the form of a ROC curve [43]. Because, in general, the best threshold of a test under examination is not known, the area under the curve (AUC) is used as a measure of performance across a range of possible thresholds (i.e., cut-offs) and identify the optimal threshold. When a perfect correlation of the predicted vs. observed progression was found, AUC was 1. Random assignment of the outcome led to an ROC AUC of 0.5. To select variables to be included in the MVA, a cut-off *p*-value = 0.10 (obtained in the UVA) was used. Regarding the MVA, a combined score was determined as the sum of scores corresponding to higher HIF-2α, eNOS, β4 integrin or CDH1 expression and lower H19 levels, based on the values above the identified cut-offs. The subsequent ROC study revealed that a value of 3 or more of “BioScore” results in a prognostic factor for PCa progression. Thus, the prognostic impact of the identified “BioScore” on progression-free survival (PFS) was analyzed using the Kaplan–Meier method and MVA using the log-rank tests to obtain a Hazard Ratio with associated 95% confidence intervals and *p*-values.

## 3. Results 

### Evaluation of H19/Cell Adhesion Molecules Circuitry on PCa Biopsies as Prognostic Markers

Previously, we demonstrated (i) a coordinated expression of eNOS/HIF-2α in about 30% of radical prostatectomy tissues from aggressive/metastatic PCa [23], (ii) a transcriptional down-regulation of lncRNA H19 under combined estrogen and hypoxia treatment in PCa cells that, in turn, induced both E-cadherin and β4 integrin expression [24], and (iii) a contribution of the above H19/cell adhesion molecules circuitry in governing in vivo tumor growth and metastasis formation [25]. 

Next, we characterize at the molecular level prostate tumors at the time of biopsies evaluating, as novel prognostic biomarkers, eNOS, HIF-2α, and β4 integrin by immunohistochemistry (IHC) and, in parallel, H19 and CDH1 RNA levels by ddPCR. IHC and ddPCR assays were performed on paraffin-embedded tissues on a retrospective cohort of PCa patients (n = 30, Table 1) enrolled at the Urology Department, Università Cattolica (Rome, Italy), to perform both PCa biopsy and prostatectomy with the following inclusion criteria: (i) clinically localized PCa at diagnosis and (ii) absence of hormone treatment/radiotherapy before surgery. The disease’s clinical progression was defined by the presence of biochemical, local, or metastatic recurrence (n = 8 out of 30, follow-up range of 3–6 years).

Sections were stained with antibodies to HIF-2α, eNOS, or β4 integrin (Figure 1A and Figure 2A), and the pathologist evaluated the quantitative assessment of scoring data in tumor tissues and adjacent normal areas.

First, HIF-2α, eNOS, or β4 integrin staining by IHC was compared in normal (NT) vs. tumoral (T) tissue samples. Both eNOS and HIF-2α showed a significantly higher expression in tumor vs. contralateral normal areas (Figure 1B), revealing their potentiality as detection biomarkers also in FFPE biopsy and not only in post-surgery tissue specimens. On the contrary, β4 integrin staining was similar between samples derived from the tumor or contralateral normal tissue (Figure S2).

Next, to evaluate the complete H19/cell adhesion molecules circuitry emerging from previous studies as novel prognostic biomarkers to predict disease progression, so comparing bad vs. good prognosis tumors, H19, and CDH1 transcripts were assessed by ddPCR and normalized to housekeeping genes (Figure 2B). The univariate and multivariate analyses explored the impact of each biomarker, including both protein levels and RNA transcripts.

To evaluate whether each biomarker (i.e., HIF-2α, eNOS, β4 integrin, CDH1, and H19, along with the classical parameters, like PSA and Gleason score) has a predictive value for disease progression, a univariate analysis was conducted using the ROC curve analysis, in particular calculating the AUC (Table 2). Based on ROC curves, a significant association of higher eNOS above cut-off expression was demonstrated in biopsies with disease progression, while the association with high β4 integrin levels was not significant, despite showing a trend in that direction (Table 2 and Figure 2C). After all, statistical significance was not achieved for the end-point progression with baseline PSA, bioptic or pathological Gleason score, HIF-2α protein expression, or CDH1 and H19 RNA levels. In addition, analysis of ROC curves revealed the optimal cut-off values for each variable with progression status (Table 2).

Of note, the modulation of each variable above or below cut-off agreed with our previous results obtained on tissue microarrays upon surgery, although not all variables reached statistical significance. Specifically, a high level of eNOS, HIF-2a, β4 integrin, and CDH1 was paralleled by low H19 in recurrent PCa [23,24,25]. Therefore, to identify patients with altered estrogen and hypoxia signaling and, in turn, H19/cell adhesion molecules circuitry early, we combined the selected variables according to their modulation, above or below cut-offs.

Importantly, multivariate Cox analysis, which considers both event and time to event, revealed HIF-2α, eNOS, β4 integrin, CDH1, and H19 as the independent prognostic factors associated with PFS. Next, all these biomarkers were included in a new combined score named “BioScore” (Table 2), defined by the presence of three factors among higher HIF-2α, eNOS, β4 integrin, or CDH1 expression and lower H19 levels; this revealed its prognostic value for progression and progression-free survival, determined using the ROC analysis (*p* = 0.003) and Kaplan–Meier curve (*p* = 0.005), respectively (Figure 2D). Here, the sensitivity and specificity of “BioScore” were 87.5 (95% CI = 47.4–97.9) and 72.7 (95% CI = 49.8–89.2), respectively. In particular, the AUC equals 0.801 and the HR (95% CI) = 0.090 (0.03–0.532), showing good separation capabilities between recurrent and non-recurrent PCa patients. Although the analyses were conducted in a limited cohort, both results exhibited statistical significance (*p* = 0.003 and 0.005, respectively). Moreover, the “BioScore” was validated in an independent retrospective cohort of PCa patients exhibiting clinical–histopathologic features similar to those of our original cohort (n = 11, age range, 63 to 77 years; PSA range, 2.4 to 20.0 ng/mL; Gleason score range, 6 (3 + 3) to 9 (4 + 5); tumor–node–metastasis range, pT2c-T3b; follow-up range 24–36 months) with the disease’s clinical progression, defined as above (n = 5 out of 11, time of recurrence range of 4–6 months). Here, the “BioScore” revealed a prognostic value for progression and progression-free survival, determined using the ROC analysis, with an area under the ROC curve (95% confidence interval) = 0.833 (0.499 to 0.976) and *p*-value = 0.0125.

## 4. Discussion

Evaluation of recurrence risk in prostate cancer remains a challenge in the field and needs further improvement. Here, we employed the potentiality of H19/cell adhesion molecules circuitry as a novel prognostic tool for aggressive and metastatic PCa. Analysis was performed on paraffin-embedded biopsies by IHC to evaluate the level of eNOS, HIF2α, and β4 integrin and by ddPCR to quantify H19 and CDH1 transcripts. Univariate analysis demonstrated an association of higher eNOS expression with progression/metastatic disease (Table 2 and Figure 2C), in agreement with previous data on surgical tissue [23] and straightening the relevance of these results, since analysis on prostatic biopsy in some cases presents limitations and often does not confirm data obtained from tissue [44]. Of note, standard clinical and histopathological parameters like baseline PSA and bioptic or pathological Gleason score showed no significant association (Table 2). Here, we identified an efficient “BioScore” resulting from a multivariate analysis of combined biomarkers defined by the presence of three factors among higher HIF-2α, eNOS, β4 integrin or CDH1 expression over cut-off and a lower H19 level under cut-off. In addition, the “BioScore” prognostic value for progression and progression-free survival, determined using the ROC analysis and Kaplan–Meier curve, revealed an excellent separation capability between recurrent and non-recurrent PCa patients (Figure 2D). These data, at least as proof of principle, revealed that the current “BioScore”, based on molecular and statistical methods, correctly identifies patients with disease progression and suggests to early identify patients with altered estrogen and hypoxia signaling with metastatic potential linked to H19/cell adhesion molecules circuitry. These results, while favoring and supporting the mechanism of the cohesive metastatic phenotype, are in agreement with recent data demonstrating upregulation of β4 integrin expression in bone metastases and during progression to castration-resistant metastatic prostate cancer ([28] and references therein). Similarly, E-cadherin expression persists in higher-grade aggressive prostate cancer [20,29], while the loss of E-cadherin signaling is observed in epithelial-to-mesenchymal transition, providing an example of tumor phenotype plasticity [20,45]. The proposed “BioScore” might represent a preoperative prognostic tool that is virtually capable of anticipating clinical outcomes at the time of biopsy. However, the possibility to routinely assess the “BioScore” might present some limitations. While protein evaluation by IHC on FFPE blocks can be easily combined with the diagnostic routine of biopsies, the use of ddPCR for diagnostic purposes could instead require advanced technology with specialized personnel in a standard pathology laboratory. In this regard, the ddPCR method presented here was initially designed for diagnostic implementation; for example, automated RNA extraction was performed with standard instrumentation for nucleic acid purification as well as custom optimized primers for ddPCR and standard enzymes for preAmp and ddPCR. In conclusion, ddPCR currently represents a promising method for diagnosing several diseases, as it is highly efficient in template detection with reduced costs and simple analysis of results that does not require a bioinformatic approach or a specialized team [46,47]. Another important aspect lies in the economic impact of these results, which are not based on high-throughput technologies, thus ensuring lower cost and rapid execution. Despite this clear advantage, cancer research is a growing field with quick development in novel diagnostic and therapeutic strategies, from enhanced cancer immunotherapy to prime editing [48,49]. The possibility of testing these emerging technologies as a complement to or enhancement of the prognostic potential of the “BioScore” would be a long-term goal for future translational research.

However, our study has inherent limitations, including the retrospective nature of the investigation, which involved a single institution with a limited number of patients, even if representative of all-day clinical practice. The relative heterogeneity of the cohort was nevertheless mitigated by standard institutional routine procedures to ensure optimized quality of biopsy material and biopsy core length. In addition, most patients (47/50) presented with relatively homogeneous clinical and pathological characteristics (ISUP 2-3), representing the most challenging group of patients in terms of prognosis at diagnosis. Further validation requires larger and multi-center cohorts as well as a machine learning approach, possibly exploring how these tools might be integrated into existing clinical workflows. In addition, standardization of methods is also needed to ensure the clinical utility of these biomarkers. Overall, this study highlights the critical role of the circuitry of H19/cell adhesion molecules in prostate cancer progression. Although results from biopsy require validation in a larger cohort, the definition of “BioScore” as a molecular signature for metastasis dissemination provides a rationale for enhanced patient stratification. This deeper molecular understanding enables a more precise classification of patients based on the underlying tumor dissemination program regulated by the hypoxia and estrogen stimuli on the H19/cell adhesion molecules circuitry and provides a rationale for novel diagnostic strategies. 

## Figures and Tables

**Table 1:** Clinical and pathological features of patients.

PCa Patients	Age	PSA (ng/mL)	Pathologic Gleason Score	Pathologic Stage	Recurrence	Time of Recurrence (Months)
PCa#1	66	11.98	7 (4 + 3)	pT2c pNx pMx	-	
PCa#2	58	5	7 (3 + 4)	pT2c pNx pMx	-	
PCa#4	56	2.15	7 (4 + 3)	pT3a pN0 pMx	yes	4
PCa#7	69	4.72	7 (4 + 3)	pT3b pN0 pMx	yes	6
PCa#8	71	14	7 (3 + 4)	pT2c pNx pMx	-	
PCa#9	69	18.5	7 (3 + 4)	pT3a pN0 pMx	yes	12
PCa#10	75	10.25	7 (3 + 4)	pT2c pNx pMx	yes	36
PCa#12	64	1.79	7 (3 + 4)	pT2c pNx pMx	-	
PCa#13	55	6.12	6 (3 + 3)	pT2 pNx pMx	-	
PCa#14	69	7.36	6 (3 + 3)	pT2c pNx pMx	-	
PCa#15	60	5.9	7 (4 + 3)	pT2c pN0 pMx	-	
PCa#16	67	7.9	7 (3 + 4)	pT2a pNx pMx	-	
PCa#18	74	6.2	7 (4 + 3)	pT2c pN0 pMx	-	
PCa#20	75	4.88	7 (3 + 4)	pT3a pN0 pMx	-	
PCa#23	75	6.3	7 (4 + 3)	pT2c pNx pMx	yes	4
PCa#31	75	16.5	7 (3 + 4)	pT2c pN0 pMx	-	
PCa#33	63	5.36	7 (3 + 4)	pT2c pNx pMx	-	
PCa#34	70	18.04	7 (3 + 4)	pT2c pN0 pMx	-	
PCa#35	68	5.75	7 (4 + 3)	pT3b pN0 pMx	yes	7
PCa#36	63	14	7 (3 + 4)	pT2c pN0 pMx	-	
PCa#40	74	8.4	7 (3 + 4)	pT2c pNx pMx	-	
PCa#41	69	6	7 (4 + 3)	pT2c pNx pMx	yes	7
PCa#42	78	15	7 (4 + 3)	pT3a pNx pMx	-	
PCa#43	67	5.5	7 (3 + 4)	pT2c pN0 pMx	-	
PCa#44	67	13.5	7 (4 + 3)	pT2c pN0 pMx	-	
PCa#45	69	11.3	9 (4 + 5)	pT3b pN0 pMx	yes	8
PCa#46	70	8.68	7 (3 + 4)	pT2c pN0 pMx	-	
PCa#48	61	18	7 (4 + 3)	pT3a pN0 pMx	-	
PCa#49	65	6.6	7 (4 + 3)	pT2c pN0 pMx	-	
PCa#50	50	11	7 (3 + 4)	pT2c pNx pMx	-	

**Table 2:** Univariate and multivariate analysis for prognostic variable identification.

Analysis	Variable (Cut-Off)	AUC (95% CI); *p*
Univariate	bGS (≥4 + 3)	0.602 (0.408–0.775); *p* = 0.401
	pGS (≥4 + 3)	0.716 (0.523–0.864); *p*= 0.059
	PSA (≥6.3)	0.568 (0.376–0.747); *p* = 0.561
	eNOS (>1)	0.853 (0.654–0.96); *p* = 0.001
	HIF-2a (>0)	0.610 (0.397–0.797); *p* = 0.355
	b4 integrin (>4)	0.693 (0.499–0.847); *p* = 0.097
	H19 (≤1.1392)	0.614 (0.149–0.784); *p* = 0.349
	CDH1 (≥0.0051)	0.636 (0.442–0.803); *p* = 0.218
Multivariate	BioScore ^§^ (≥3)	0.801 (0.616–0.923); *p* = 0.003

AUC = area under ROC curve; CI = confidence interval. bGS = bioptic Gleason Score. pGS = pathological Gleason score. ^§^ Three among eNOS (>1), HIF-2α (>0), β4 integrin (>4), H19 (≤1.1392), and CDH1 (≥0.0051).

## Data Availability

The data underlying this article are available from the corresponding author on reasonable request.

## Supplementary Materials

The following supporting information can be downloaded at https://www.mdpi.com/article/10.3390/biomedicines12102322/s1, Figure S1: Set up of ddPCR analysis in PCa biopsies; Figure S2: β4 integrin expression by IHC on PCa biopsies and score in normal and tumoral areas.

## List of Abbreviations

ADT	androgen-deprivation therapy
CRPC	castration-resistant prostate cancer
EMT	epithelial-to-mesenchymal transition
eNOS	endothelial Nitric Oxide Synthase
ER	epitope retrieval
H&E	Hematoxylin and Eosin staining
HIF-2a	Hypoxia-Inducible Factor 2 Alpha
lncRNA	long noncoding RNA
MVA	multivariate analysis
NGS	next-generation sequencing
PCa	prostate cancer
PSA	prostate-specific antigen
UVA	univariate analysis
