# Supplementary material for: Prognostic Impact of H19/Cell Adhesion Molecules Circuitry on Prostate Cancer Biopsy
Source: Biomedicines. 2024 Oct 12;12(10):2322. doi: 10.3390/biomedicines12102322 (PMC11504315; doi:10.3390/biomedicines12102322)
Supplement: Supplementary file 1 [file biomedicines-12-02322-s001.zip › biomedicines-3226815-supplementary.pdf]

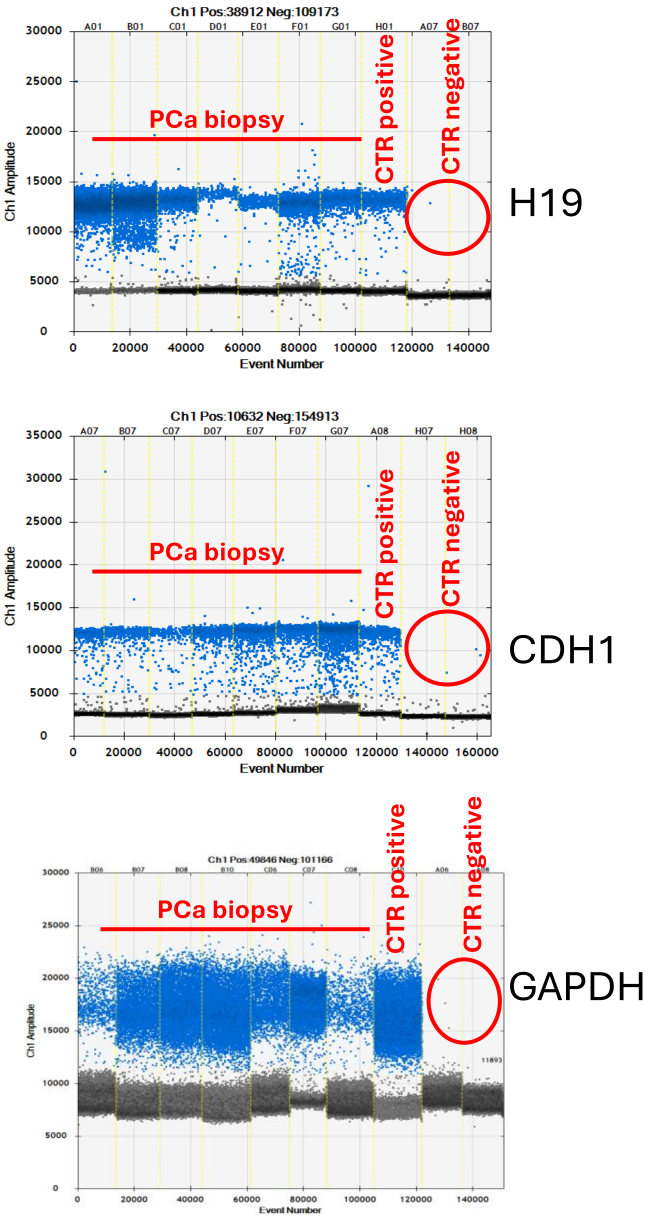

Figure S1. Set up of ddPCR analysis in PCa biopsies. PCa biopsy samples were analyzed by ddPCR as described in methods. Briefly, RNA was extracted from two FFPE slices (3-4µm) using automated instrument for nucleic acid extraction and resuspended in 20 microliter of DEPC-H_2O. 10 microliter were subjected to retrotranscripiton with high capacity kit (Applied Biosystems). PreAmp step were performed using 2 microliter of cDNA, EvaGreen Taq and specific primers (40nM final concentration). 2 microliter of 1:10 dilution of preAmp was used to perform ddPCR using EvaGreen as manufacturer's instruction. Quantification of each gene was expressed in copy number/microliter. Panels are representative experiments for H19, CDH1 and GAPDH detection by ddPCR in several PCa biopsy samples, positive and negative control are showed.

A

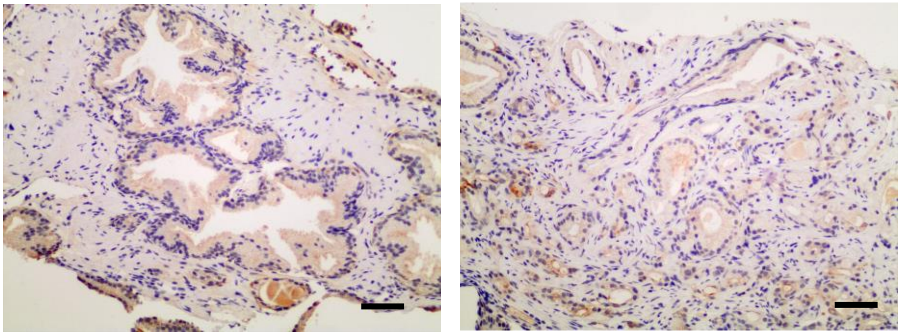

B

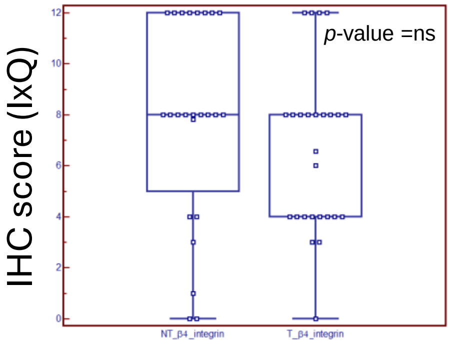

Figure S2. β4 integrin expression by IHC on PCa biopsies and score in normal and tumoral areas. A) Representative IHC staining of PCa biopsies with a specific antibody to β4 integrin in normal (left) and tumoral (right) areas. Scale bar (210 µm) is showed as black line. B) The IHC score was evaluated as Intensity x quantity (IxQ) in normal (NT) and tumoral (T) areas for β4 integrin staining. ns = non significant.
